# Interaction between Bombyx mori Cytoplasmic Polyhedrosis Virus NSP8 and BmAgo2 Inhibits RNA Interference and Enhances Virus Proliferation

**DOI:** 10.1128/spectrum.04938-22

**Published:** 2023-06-21

**Authors:** Jun Pan, Qunnan Qiu, Dhiraj Kumar, Jian Xu, Xinyu Tong, Zeen Shen, Min Zhu, Xiaolong Hu, Chengliang Gong

**Affiliations:** a School of Biology and Basic Medical Sciences, Soochow University, Suzhou, Jiangsu, China; b Agricultural Biotechnology Research Institute, Agricultural Biotechnology and Ecological Research Institute, Soochow University, Suzhou, China; c Department of Zoology, Hansraj College, University of Delhi, Delhi, India; Technion—Israel Institute of Technology

**Keywords:** *Bombyx mori* cytoplasmic polyhedrosis virus, nonstructural protein 8, Argonaute 2, suppression of RNAi

## Abstract

Some insect viruses encode suppressors of RNA interference (RNAi) to counteract the antiviral RNAi pathway. However, it is unknown whether Bombyx mori cytoplasmic polyhedrosis virus (BmCPV) encodes an RNAi suppressor. In this study, the presence of viral small interfering RNA (vsiRNA) in BmN cells infected with BmCPV was confirmed by small RNA sequencing. The Dual-Luciferase reporter test demonstrated that BmCPV infection may prevent firefly luciferase (Luc) gene silencing caused by particular short RNA. It was also established that the inhibition relied on the nonstructural protein NSP8, which suggests that NSP8 was a possible RNAi suppressor. In cultured BmN cells, the expressions of viral structural protein 1 (*vp1*) and NSP9 were triggered by overexpression of *nsp8*, suggesting that BmCPV proliferation was enhanced by NSP8. A pulldown assay was conducted with BmCPV genomic double-stranded RNA (dsRNA) labeled with biotin. The mass spectral detection of NSP8 in the pulldown complex suggests that NSP8 is capable of direct binding to BmCPV genomic dsRNA. The colocalization of NSP8 and *B. mori* Argonaute 2 (BmAgo2) was detected by an immunofluorescence assay, leading to the hypothesis that NSP8 interacts with BmAgo2. Coimmunoprecipitation further supported the present investigation. Moreover, vasa intronic protein, a component of RNA-induced silencing complex (RISC), could be detected in the coprecipitation complex of NSP8 by mass spectrum analysis. NSP8 and the mRNA decapping protein (Dcp2) were also discovered to colocalize to processing bodies (P bodies) for RNAi-mediated gene silencing in Saccharomyces cerevisiae. These findings revealed that by interacting with BmAgo2 and suppressing RNAi, NSP8 promoted BmCPV growth.

**IMPORTANCE** It has been reported that the RNAi pathway is inhibited by binding RNAi suppressors encoded by some insect-specific viruses belonging to *Dicistroviridae*, *Nodaviridae*, or *Birnaviridae* to dsRNAs to protect dsRNAs from being cut by Dicer-2. However, it is unknown whether BmCPV, belonging to *Spinareoviridae*, encodes an RNAi suppressor. In this study, we found that nonstructural protein NSP8 encoded by BmCPV inhibits small interfering RNA (siRNA)-induced RNAi and that NSP8, as an RNAi suppressor, can bind to viral dsRNAs and interact with BmAgo2. Moreover, vasa intronic protein, a component of RISC, was found to interact with NSP8. Heterologously expressed NSP8 and Dcp2 were colocalized to P bodies in yeast. These results indicated that NSP8 promoted BmCPV proliferation by binding itself to BmCPV genomic dsRNAs and interacting with BmAgo2 through suppression of siRNA-induced RNAi. Our findings deepen our understanding of the game between BmCPV and silkworm in regulating viral infection.

## INTRODUCTION

RNA interference (RNAi) widely exists in eukaryotes (1–5). The RNAi pathway plays an important role in the antiviral defense mechanism of insects (6). Previously, studies have shown a model of the exogenous RNA interference (exo-RNAi) pathway in which a viral genome consisting of double-stranded RNA (dsRNA) is cleaved by Dicer-2 (Dcr-2) into viral small interfering RNAs (vsiRNAs) (7). These duplexes of ~21-nucleotide (nt) small RNAs with a characterized signature of 2-nt 3′ overhangs are the most efficient triggers to mediate the sequence-specific degradation of target mRNA (8), by being incorporated into RNA-induced silencing complex (RISC) via interaction with Argonaute 2 (Ago2) (9). One of the strands of the vsiRNAs (the passenger strand) is eliminated, while the other strand (the guide strand) is still retained in RISC to direct endonucleolytic cleavage of the viral target RNA upon recognition of a fully complementary sequence (10).

Deep sequencing data have shown that several small RNAs are present in both *Drosophila* and mosquito cells that are infected with various viruses (11–13). The length distribution of these small RNAs is approximately17 to 35 nt, and they directly map to different segments of the viral genome (14). Moreover, the viral genomic dsRNAs could be recognized and digested into small interfering RNA (siRNA) duplexes by cytoplasmic RNase III class enzyme Dcr-2 (15). However, these vsiRNAs generated in host cells create high matching areas (hot spots) and low coverage areas (or no matching areas, cold spots) with certain viral genome regions (16). Moreover, a previous study showed that for the viral genomes of two viruses belonging to different taxonomic species, *Homalodisca coagulate* virus 1 and *Homalodisca vitripennis* reovirus, RNAs could also be cleaved by Dcr-2 in host cells, but the distribution characteristics of vsiRNAs of different viruses are inconsistent in their viral genomes (17). According to earlier research, when RNA viruses infect host cells, the exo-RNAi pathway is activated, and the host cells exploit this route as an antiviral defense mechanism to stop the virus from multiplying (7).

Further, RNAi-inhibiting proteins termed RNAi suppressors encoded by viruses have been discovered. Some of these proteins could inhibit RNAi by binding to viral dsRNA genomes and protect viral dsRNA from being cut by Dcr-2 enzyme in the infected host cells, including B2, DCV-1A, and VP3, encoded by flock house virus (FHV), *Drosophila* C virus (DCV) and Culex Y virus (CYV), respectively (18–20). It was found that FHV B2 could bind to vsiRNA to prevent vsiRNA binding to RISCs (21). Recent research has demonstrated that the CrPV-1A protein expressed by the cricket paralysis virus (CrPV) and the VP1 protein encoded by the Nora virus (NV) can decrease Ago2 protein function (22, 23).

Bombyx mori cytoplasmic polyhedrosis virus (BmCPV) belongs to the family *Reoviridae*. The genome of BmCPV consists of 10 dsRNA segments (S1 to S10) (24). Previous studies indicated that viral structural proteins VP1, VP2, VP3, VP4, VP6, and VP7 were encoded by BmCPV genomic dsRNA segments S1, S2, S3, S4, S6, and S7, respectively, and that viral nonstructural proteins NSP5, NSP8, NSP9, and polyhedrin were encoded by segments S5, S8, S9, and S10, respectively. VP1 is a major capsid protein (25). VP2 is an RNA-dependent RNA polymerase, which is mainly responsible for the transcription and replication of viral RNA (25, 26). VP3 is a spike protein with an RGD motif. It has been suggested that cell entry of BmCPV depends on the binding of VP3 to the host cell surface receptor (27). VP4 is a viral turret protein with RNA guanylyltransferase, 7-*N*-methyltransferase, and 2′-*O*-methyltransferase activities which catalyzes the transcriptional activation and capping of viral mRNA (28). A recent study indicated that PINK1-Parkin-mediated mitophagy is induced by the interaction of VP4 with host Tom40 (29). VP5 is a minor capsid (25). VP7 is a large project protein, which is cleaved to form a structural protein of 40 kDa between Asn291 and Ala292 (26). Moreover, it has been reported that the cell entry of BmCPV can be promoted by the interaction of VP7 with tight junction protein claudin-2 (30). BmCPV can produce polyhedra, a polyhedrin crystal, in the cytoplasm of infected cells. Many viral particles are embedded into polyhedra, which can protect the virus from destruction by the harsh environment (31, 32). There are few studies on NSPs except for polyhedrin. Moreover, small RNA sequencing indicated that vsiRNAs can be produced by RNAi from viral genomic dsRNAs (16, 33). The game between the virus and the host determines whether the host is infected and its pathogenesis. To date, it is not known whether BmCPV encodes an RNAi suppressor to counteract the host's defense response.

In this study, we discovered that BmCPV genomic dsRNAs may be cleaved into vsiRNA, that NSP8 inhibits siRNA-induced RNAi, and that NSP8, as an RNAi suppressor, can bind to viral dsRNAs and interact with *B. mori* Ago2 (BmAgo2). Moreover, vasa intronic protein was found to be coprecipitated with NSP8. Heterologously expressed NSP8 was localized to processing bodies (P bodies) in Saccharomyces cerevisiae. These findings suggested that NSP8 enhances BmCPV proliferation by interacting with BmAgo2 through suppression of siRNA-induced RNAi.

## RESULTS

### BmCPV RNA could be cleaved to small RNAs in the infected BmN cells.

**FIG 1:**
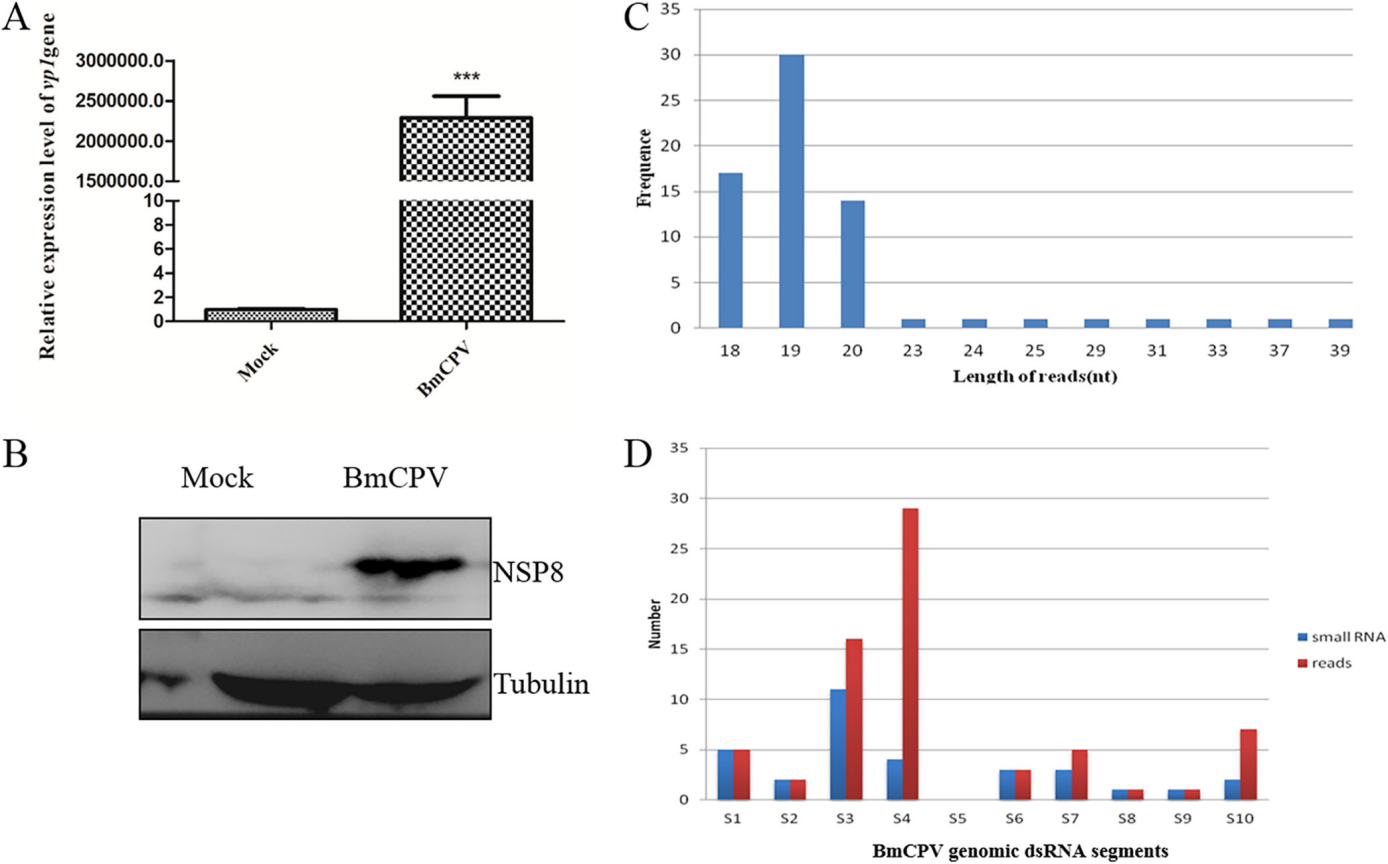
Viral small RNAs derived from BmCPV in infected BmN cells. A total of 10^7^ BmN cells were infected with BmCPV (MOI of 3), and the cells collected at 144 h postinfection were used to determine BmCPV *vp1* gene expression by qRT-PCR, NSP8 protein expression by Western blotting, and viral small RNAs by small RNA sequencing. (A) BmCPV *vp1* gene expression determined by qRT-PCR in infected BmN cells. Mock, noninfected BmN cells; BmCPV, BmCPV-infected BmN cells (MOI of 3) at 144 h postinfection. The *TIF-4A* gene was used as an internal control. (B) BmCPV NSP8 expression detected by Western blotting with anti-NSP8 antibody. Mock, noninfected BmN cells; BmCPV, BmCPV-infected BmN cells at 144 h postinfection. Tubulin was used as an internal reference. (C) Length distribution of viral small RNAs produced in BmN cells. (D) Distribution of viral small RNA on the genomic dsRNA segments.

Quantitative real-time PCR (qRT-PCR) analysis of the inoculated cells revealed that BmCPV infection raised the expression level of the BmCPV *vp1* gene (Fig. 1A), indicating that the BmN cells had acquired BmCPV infection. To detect BmCPV NSP8 expression, the mouse anti-NSP8 antibody was prepared by immunizing mice with recombinant NSP8 expressed in Escherichia coli. The particular signal band indicating NSP8 was recognized only in the midgut of BmCPV-infected silkworm larvae and the transformed E. coli with pET-28a-nsp8 (see Fig. S1 in the supplemental material), indicating that the anti-NSP8 antibody produced may be employed to detect NSP8 expression. In this study, a specific signal band representing NSP8 was detected in the BmCPV-infected BmN cells, confirming that the cultured BmN cells were infected by BmCPV (Fig. 1B). As a result, the BmCPV-infected BmN cells at 6 days postinfection were utilized for small RNA sequencing to investigate whether small RNAs were generated from BmCPV RNA.

Following the removal of low-quality reads, 61,237,540 clean reads were recovered. The clean reads were mapped to the BmCPV viral genome and the silkworm reference genome, 69 and 37,145,733 reads were found mapped to the BmCPV genome and silkworm genome, respectively, indicating that the vsiRNAs could be generated from BmCPV RNAs. The sequences and abundances of the vsiRNAs are listed in Table S4. Thirty-one vsiRNAs were found in the BmCPV-infected BmN cells, and all of vsiRNAs were derived from the sense strand of BmCPV genomic dsRNAs. The length distribution of vsiRNA was examined, and the results revealed that vsiRNAs ranged in length from 18 to 39 nt, with most of them falling between 19 and 20 nt. The numbers of vsiRNAs with lengths of 18, 19, and 20 nt were 17, 30, and 14, respectively, and the read count for other vsiRNAs with lengths of 23, 24, 25, 29, 31, 33, 37, and 39 nt was one each (Fig. 1C). Moreover, the distribution of vsiRNAs in the BmCPV genomic dsRNA segment was also analyzed, and the results showed that the number of vsiRNAs that mapped to the BmCPV genomic dsRNA S3 segment was the largest, i.e., 11, followed by 5 for the S1 segment and 4 for the S4 segment; the vsiRNA generated by the S5 segment was not found (Fig. 1D). According to these findings, the BmCPV RNA in the infected cells could be cleaved into short or small RNAs.

### BmCPV infection inhibits siRNA-induced RNAi in the cultured BmN cells.

**FIG 2:**
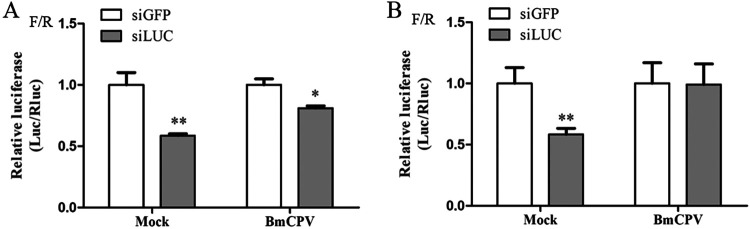
Effects of BmCPV infection on siRNA-induced RNAi. BmN cells (10^5^ cells in 1 mL of medium) were inoculated with BmCPV (MOI of 3) for 48 h (A) and 72 h (B), and then the cells were cotransfected with the firefly luciferase gene reporter plasmid pGL3^OTU^ (2 μg), *Renilla* luciferase gene reporter plasmid pRL-TK (0.2 μg), and siRNA (1 μg); after transfection for 60 h, the cells were collected and lysed. One hundred micrograms of total cell protein was used to determine the activity of firefly and *Renilla* luciferases, and then the ratio of the firefly luciferase activity to *Renilla* luciferase activity (F/R ratio) was investigated. Mock, noninfected BmN cells; BmCPV, BmN cells infected with BmCPV (MOI of 3) for 48 h (A) and 72 h (B) before transfection. siLUC, cells were treated with LUC-siRNA; siGFP, cells were treated with GFP-siRNA; F/R, ratio of firefly luciferase activity to *Renilla* luciferase activity. **, *P* < 0.01; *, *P* < 0.05.

To explore whether the RNAi pathway was inhibited by BmCPV infection, normal BmN cells and BmN cells infected with BmCPV were, respectively, cotransfected with pGL3^OTU^, pRL-TK, and the siRNA (siRNA-luciferase [siLUC] or siRNA-green fluorescent protein [siGFP]) at 48 h and 72 h postinfection. The ratio of firefly luciferase activity to Renilla luciferase activity (F/R ratio) was used to assess inhibition of the RNAi pathway by BmCPV infection. An examination of the data indicated that the F/R ratio for the BmN-siLUC group was much lower than that for the BmN-siGFP group, indicating that the firefly luciferase gene was preferentially silenced by LUC-siRNA (Fig. 2A and B). The F/R ratio for the CPV-siLUC group, for which the BmCPV-infected cells were treated with siLUC at 48 h postinfection, was also markedly lower than that that for the CPV-siGFP group, for which the BmCPV-infected cells were treated with siGFP, suggesting that firefly luciferase gene expression could be inhibited by treatment with siLUC regardless of whether the cells were infected with BmCPV. However, the F/R ratio for the CPV-siLUC group was significantly higher than that for the BmN-siLUC group (Fig. 2A). At 72 h postinfection, no significant difference in F/R ratio was found between the CPV-siLUC and CPV-siGFP groups, but the F/R ratio for the CPV-siLUC group was higher than that for the BmN-siLUC group (Fig. 2B). In addition, we investigated the production of small RNAs in the midgut during different stages of BmCPV infection, and the results showed that the relative level of detected small RNAs generally decreased with virus infection, indicating that the production of small RNAs was suppressed due to virus infection (Fig. S3). These findings suggest that BmCPV infection may suppress siRNA-induced RNAi in cultured BmN cells.

### BmCPV NSP8 suppresses siRNA-induced RNAi in BmN cells.

**FIG 3:**
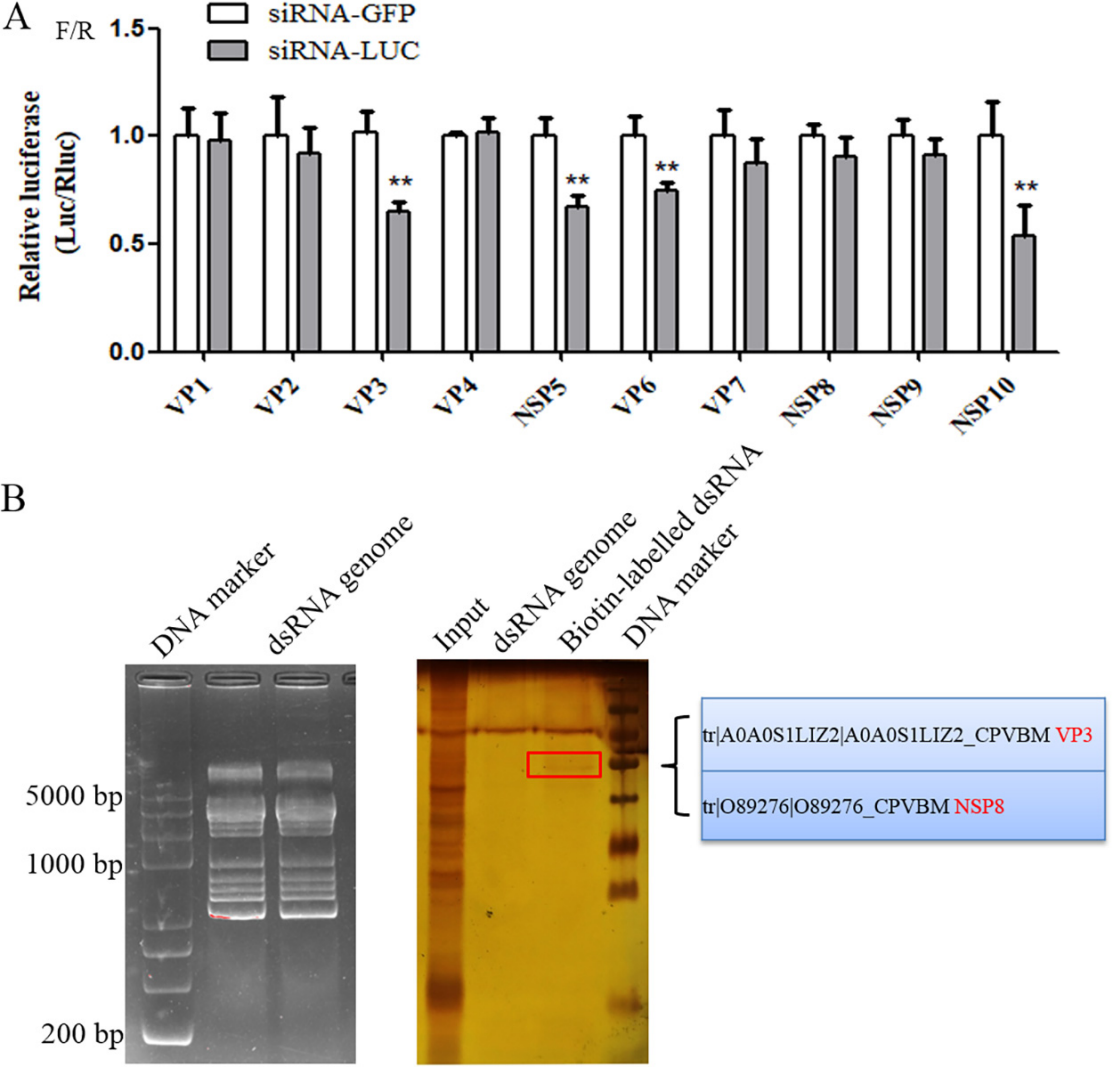
Effect of BmCPV genes on siRNA-induced RNAi in BmN cells. (A) Effect of BmCPV genes on siRNA-induced RNAi. The pIZT-CS1, pIZT-CS2, pIZT-CS3, pIZT-CS4, pIZT-CS5, pIZT-CS6, pIZT-CS7, pIZT-CS8, pIZT-CS9, and pIZT-CS10 vectors (2 μg) expressing BmCPV VP1, VP2, VP3, VP4, NSP5, VP6, VP7, NSP8, NSP9, and NSP10 (polyhedrin), respectively, were cotransfected with pGL3^OTU^ (2 μg), pRL-TK (0.2 μg), and the siRNA (siLUC or siGFP) (1 μg) into 10^6^ BmN cells. At 60 h after transfection, the cells were collected and used to determine the F/R ratio. (B) BmCPV dsRNA pulldown assay and LC-MS/MS identification. Left, Electrophoresis of BmCPV genomic dsRNAs; right, biotin-labeled BmCPV genomic dsRNAs were used to screen viral proteins that bind dsRNAs by using a dsRNA pulldown assay. Meanwhile, the dsRNA pulldown assay conducted with unlabeled BmCPV genomic dsRNAs was used as a control. The obtained precipitates were separated by SDS-PAGE. After the protein bands were visualized with silver staining, the differential protein bands were identified by LC-MS/MS.

**FIG 4:**
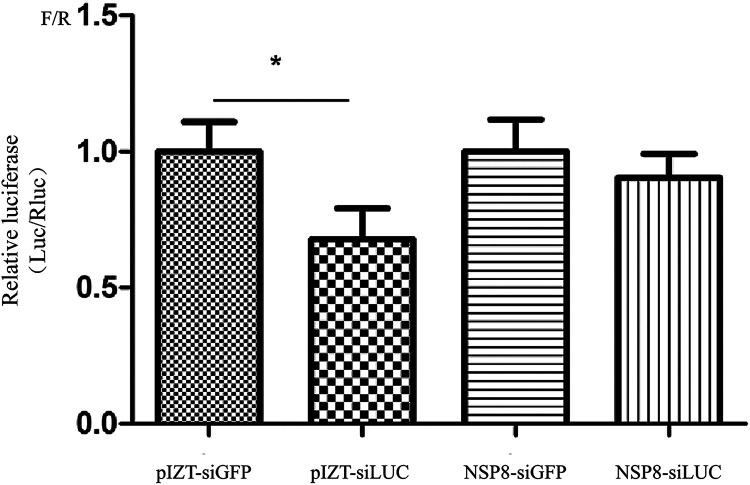
Effects of NSP8 on siRNA-induced RNAi. BmN-NSP8 and BmN-null transformed cells (10^5^ cells in 1 mL of medium) were cotransfected with a mixture of plasmid pGL3^OTU^ (2 μg), pRL-TK (0.2 μg), and siRNA (1 μg), and 60 h after transfection, the cells were collected and lysed. The total cell proteins (100 μg) were used to determine the F/R ratio. pIZT-siLUC, BmN-null transformed cells treated with LUC-siRNA; pIZT-siGFP, BmN-null transformed cells treated with GFP-siRNA; NSP8-siLUC, BmN-NSP8 transformed cells treated with LUC-siRNA; NSP8-siGFP, BmN-NSP8 transformed cells treated with GFP-siRNA. *, *P* < 0.05.

To identify which proteins encoded by BmCPV can inhibit the siRNA-induced RNAi, the constructed BmCPV protein expression vectors (pIZT-CS1, pIZT-CS2, pIZT-CS3, pIZT-CS4, pIZT-CS5, pIZT-CS6, pIZT-CS7, pIZT-CS8, pIZT-CS9, and pIZT-CS10) were cotransfected with pGL3^OTU^, pRL-TK, and the siRNA (siLUC or siGFP) into BmN cells, respectively. The cells were utilized to measure the F/R ratio after 60 h of transfection, and the findings revealed that there was no discernible change in F/R ratio between the siLUC and siGFP groups for which the BmN cells were transfected with pIZT-CS1, pIZT-CS2, pIZT-CS4, pIZT-CS7, pIZT-CS8, and pIZT-CS9, suggesting that viral proteins VP1, VP2, VP4, VP7, NSP8, and NSP9 had potential inhibitory activity for the siRNA-induced RNAi (Fig. 3A). However, the F/R ratios for the siLUC groups for which the BmN cells were transfected with pIZT-CS3, pIZT-CS5, pIZT-CS6, and pIZT-CS10 were lower than those for the corresponding siGFP groups, suggesting that viral proteins VP3, NSP5, VP6, and polyhedrin had no inhibitory activity for the siRNA-induced RNAi (Fig. 3A). A previous study showed that some viral proteins could inhibit RNAi by binding themselves to viral dsRNA genomes (18). To further determine which viral proteins are siRNA-induced RNAi suppressors, biotin-labeled BmCPV genomic dsRNA was applied to screen viral proteins that bind dsRNAs by use of a BmCPV dsRNA pulldown assay. NSP8 and VP3 were identified in the pulldown precipitation complex by liquid chromatography-tandem mass spectrometry (LC-MS/MS) (Fig. 3B). Because VP3 had no inhibitory activity for the siRNA-induced RNAi, we supposed that NSP8 was an siRNA-induced RNAi suppressor which could directly bind to BmCPV genomic dsRNAs. To further validate that NSP8 was an siRNA-induced RNAi suppressor, BmN-null and BmN-NSP8 transformed cells were, respectively, cotransfected with a mixture of pGL3^OTU^, pRL-TK, and siGFP/siLUC to determine the F/R ratio using the Dual-Luciferase reporter assay. There was no significant difference in F/R ratio between the siGFP group and the siLUC group, suggesting that siRNA-induced RNAi was inhibited by NSP8 (Fig. 4). In addition, the effect of overexpression of NSP8 on the production of small RNAs in BmCPV-infected BmN cells transfected with pIZT-NSP8 was evaluated. The results showed that the production of small RNAs was reduced in BmCPV-infected BmN cells overexpressing NSP8, indicating that NSP8 is an inhibitor of RNAi (Fig. S4).

### NSP8 promotes BmCPV gene expression.

**FIG 5:**
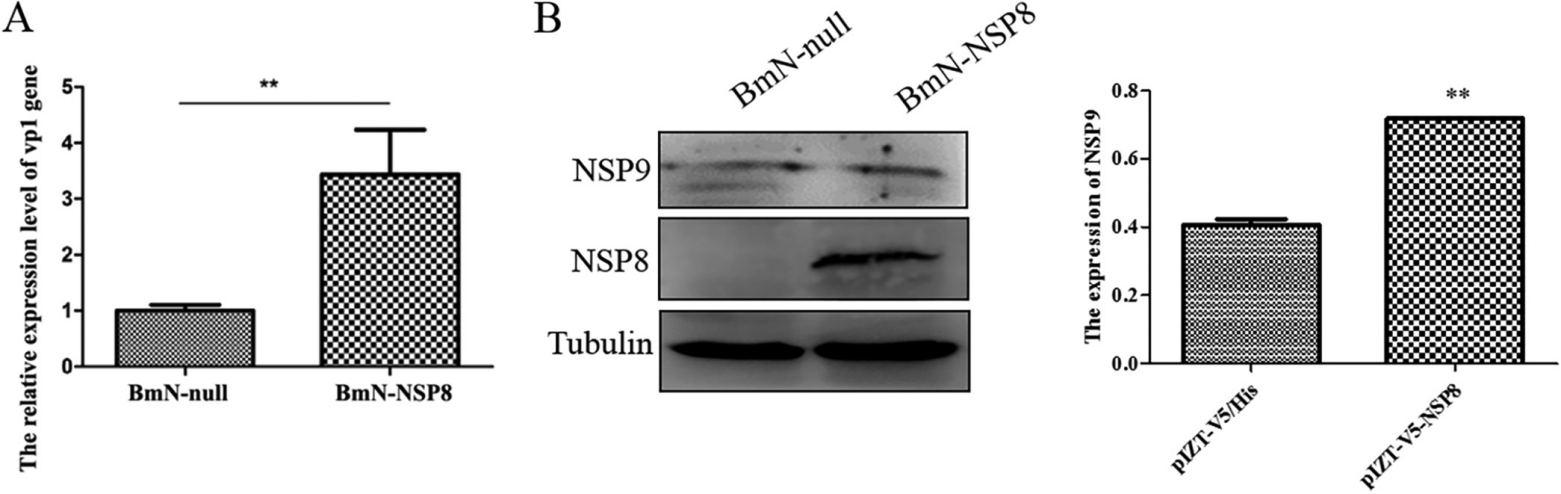
Effect of overexpressing BmCPV *nsp8* gene on viral gene expression. (A) Effects of overexpression of the *nsp8* gene on the expression level of the BmCPV *vp1* gene. A total of 10^6^ BmN-null and BmN-NSP8 transformed cells were infected with BmCPV (MOI of 3), the total RNAs were extracted from the cells at 72 h postinfection, and after the RNAs were reverse transcribed into cDNA, the relative expression level of the BmCPV *vp1* gene to the *TIF-4A* gene was determined by real-time PCR. (B) Effects of overexpression of the *nsp8* gene on the expression level of BmCPV NSP9. A total of 10^6^ BmN-null and BmN-NSP8 transformed cells were infected with BmCPV (MOI of 3), and the cells collected at 72 h postinfection were detected by Western blotting with mouse anti-NSP9 antibody or anti-NSP8 antibody. Tubulin was used as an internal reference. Left, image of the Western blot; right, the relative grayscale value of signal band NSP9 to tubulin. pIZT-V5/His, BmN-null transformed cells; pIZT-V5-NSP8, BmN-NSP8 transformed cells. **, *P* < 0.01.

To understand the function of NSP8, the effect of overexpression of the *nsp8* gene on BmCPV proliferation was investigated. Real-time PCR was used to assess the expression level of the viral structural protein *vp1* gene 72 h after BmCPV infection in BmN-null and BmN-NSP8 transformed cells. The results revealed that the BmN-NSP8 transformed cells, which express NSP8, had significantly higher levels of *vp1* gene expression than the BmN-null cells (Fig. 5A). The expression level of NSP9 at 72 h postinfection was detected by Western blotting with mouse anti-NSP9 antibody. The results showed that the expression level of NSP9 protein in BmN-NSP8 cells was obviously higher than that in BmN-null cells (Fig. 5B), indicating that NSP8 protein promoted BmCPV virus gene expression.

### Interaction of NSP8 with BmAgo*2*.

**FIG 6:**
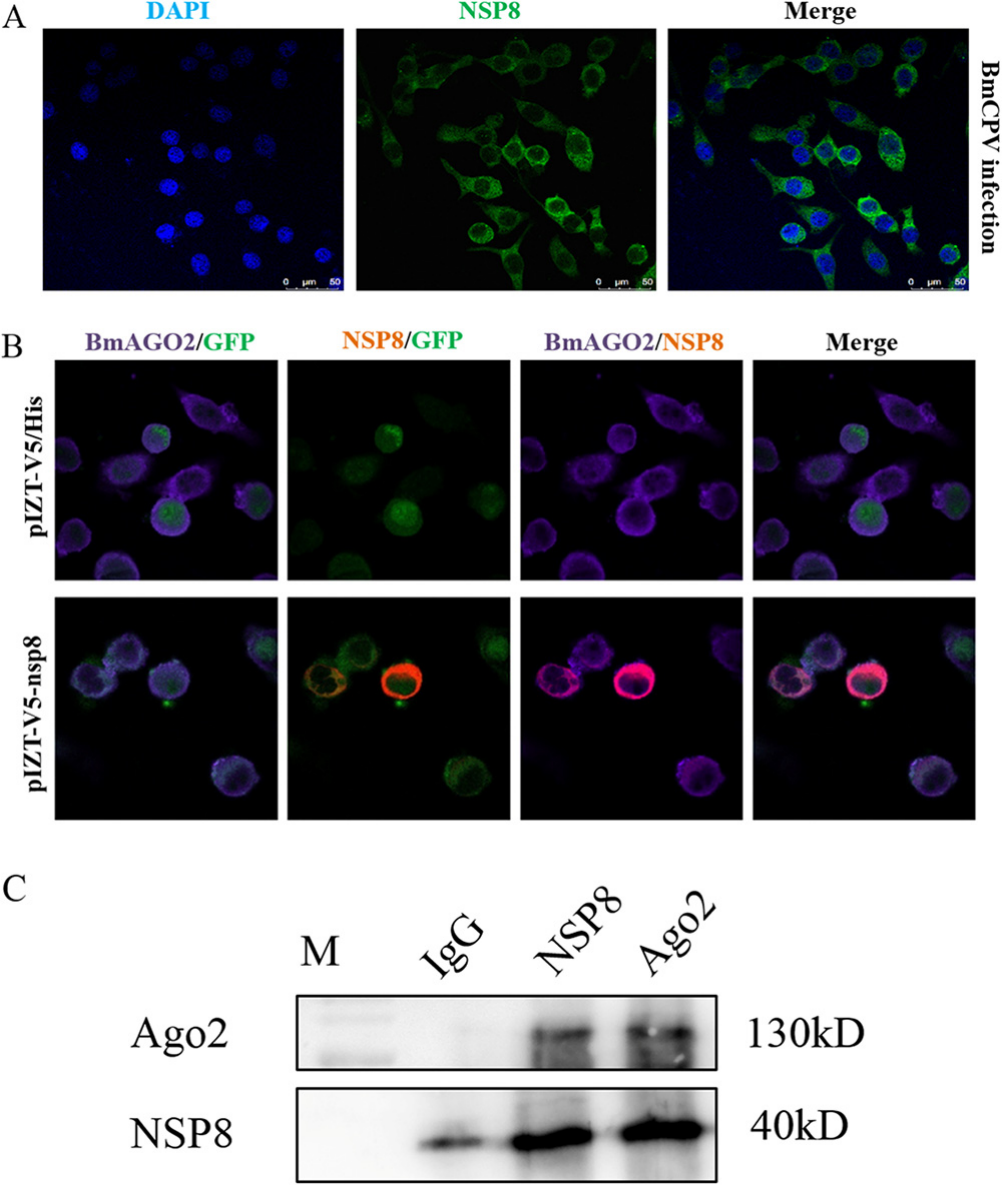
Interaction between NSP8 and BmAgo2. (A) Subcellular localization of NSP8 in BmCPV-infected BmN cells. BmCPV (MOI of 3)-infected BmN cells at 72 h postinfection were fixed with 4% paraformaldehyde for 5 min. An immunofluorescence assay was carried out using mouse anti-NSP8 antibody as the primary antibody and FITC-conjugated goat anti-mouse IgG(H+L) as the secondary antibody. The cell nucleus was labeled with DAPI (1:1,000). (B) Colocalization of NSP8 with BmAgo2. A total of 10^6^ of BmN-NSP8 and BmN-null transformed cells were fixed with 4% paraformaldehyde for 5 min. An immunofluorescence assay was carried out using anti-V5 rabbit polyclonal antibody and mouse anti-BmAgo2 antibody as the primary antibodies and using Cy3-conjugated goat anti-rabbit IgG(H+L) and Cy5-conjugated goat anti-mouse IgG as the secondary antibodies. The cell nucleus was stained with DAPI. The transformed BmN cells expressed GFP (green). The cells were observed under a confocal microscope. BmAGO2/GFP, merged BmAGO2 and GFP channels; NSP8/GFP, merged NSP8 and GFP channels; BmAGO2/NSP8, merged BmAGO2 and NSP8 channels; Merge, merged BmAGO2, GFP, and NSP8 channels. (C) Western blot detection of immunoprecipitation complexes obtained with anti-NSP8/anti-BmAgo2 antibodies. A total of 10^6^ BmCPV (MOI of 3)-infected BmN-null cells at 72 h postinfection were used to extract total protein for co-IP with 5 μL anti-NSP8/anti-BmAgo2 antibodies, and after the obtained immunoprecipitation complexes were separated by 10% SDS-PAGE, Western blotting was carried out with anti-BmAgo2/anti-NSP8 antibodies.

To characterize the distribution of NSP8 in the BmCPV-infected BmN cells, a cell immunofluorescence assay was performed. Initially, we incubated BmCPV-infected BmN cells with mouse anti-NSP8 antibody. The results showed that the green fluorescence representing NSP8 was located in the cytoplasm (Fig. 6A). Then, we used rabbit anti-V5 polyclonal antibody as the primary antibody and Cy3-conjugated goat anti-rabbit IgG(H+L) (red) as the secondary antibody, the cell nucleus was stained with DAPI (4′,6-diamidino-2-phenylindole; blue), and the result observed was the interaction of NSP8 with BmAgo2 in the BmN-NSP8 transformed cells (Fig. 6B).

To detect BmAgo2, anti-BmAgo2 antibody was prepared by immunizing mice with the recombinant truncated BmAgo2 protein expressed in E. coli. The prepared anti-BmAgo2 antibody was used to detect the recombinant truncated BmAgo2 protein expressed in E. coli BL21 transformed with pET28a-BmAgo2 and the endogenous BmAgo2 expressed in BmN cells by Western blotting. A specific signal band could be detected (Fig. S2), indicating that the prepared anti-BmAgo has specificity. According to immunofluorescence analysis, the red fluorescence (Cy3) representing NSP8 protein could be found only in the BmN-NSP8 transformed cells, whereas the purple fluorescence (Cy5) representing BmAgo2 protein could be found in both BmN-null and BmN-NSP8 transformed cells. Additionally, the signal representing NSP8 overlapped with that of BmAgo2 in the BmN-NSP8 cells, suggesting that NSP8 was colocalized with BmAgo2 protein (Fig. 6B). Coimmunoprecipitation (co-IP) was performed using anti-NSP8 or BmAgo2 to validate the interaction between NSP8 and BmAgo2. NSP8 may have interacted with BmAgo2, since BmAgo2 was identified in the coprecipitation complex of NSP8 and NSP8 was discovered in the coprecipitation complex of BmAgo2 (Fig. 6C).

### NSP8 colocalized with Dcp2 in P bodies of yeast.

**FIG 7:**
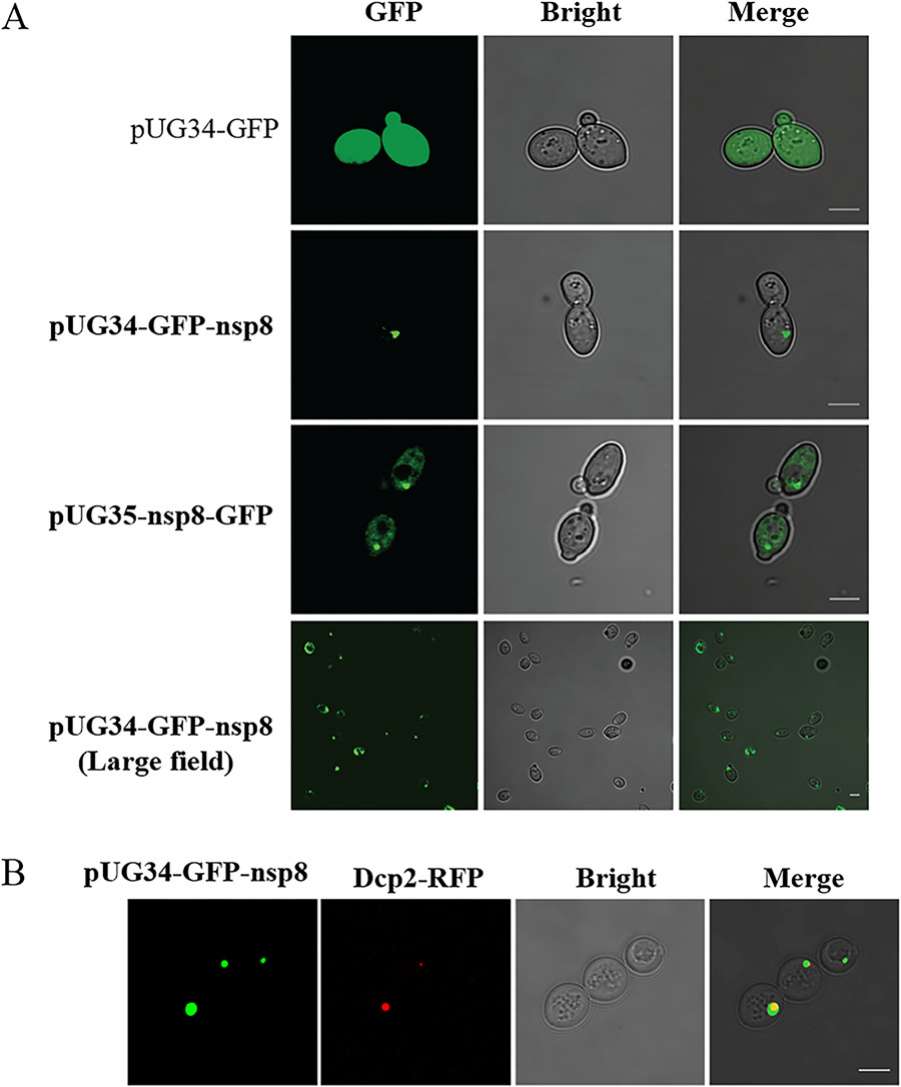
Localization of recombinant NSP8 in yeast. (A) Recombinant NSP8 localized in the cytoplasm of yeast. A total of 10^7^ cells of yeast strain BY4743 were transfected with 2 μg pUG34-GFP-nsp8/pUG35-nsp8-GFP, and the positive transformants were induced in MY liquid medium without methionine to express recombinant GFP-NSP8/NSP8-GFP for 1 h. The yeast cells were observed under a Zeiss Imager confocal microscope, and pUG34-GFP-transformed cells were used as a control. (B) Colocalization of recombinant NSP8 with Dcp2 protein in yeast. A total of 10^7^ yeast cells were cotransfected with 2 μg of the pUG34-GFP and 2 μg of the plasmid encoding the mRNA decapping protein fused with red fluorescent protein (Dcp2-RFP). After 12 h, the positive transformants were induced to express Dcp2-RFP for 1 h, and the cells were observed under a Zeiss Imager confocal microscope.

Yeast, a model organism, has been reported to be used to examine the subcellular localization of green algal plasma membrane H^+^-ATPases (34) and spore wall protein NbSWP12 of the microsporidium Nosema bombycis (35). To further understand the function of NSP8, the yeast strain BY4743 was used to characterize the distribution of recombinant NSP8 in the cell. The results showed that GFP was evenly distributed in the transfected yeast with plasmid pUG34-GFP, and both recombinant GFP-nsp8 and nsp8-GFP proteins were aggregated in the cytoplasm of yeast (Fig. 7A). P bodies are ribonucleoprotein (RNP) granules located in the cytoplasm. DCP is a marker for P bodies (36). The previous study indicated that BmAgo1 interacts with *B. mori* Dcp2 and that BmAgo1 functions in the microRNA (miRNA)-mediated RNAi pathway (37). In this study, GFP-nsp8 was found to be colocalized with Dcp2-red fluorescent protein (Dcp2-RFP), and therefore, we suggest that NSP8 was localized at the P body (Fig. 7B).

### Candidate interacting proteins of NSP8.

**FIG 8:**
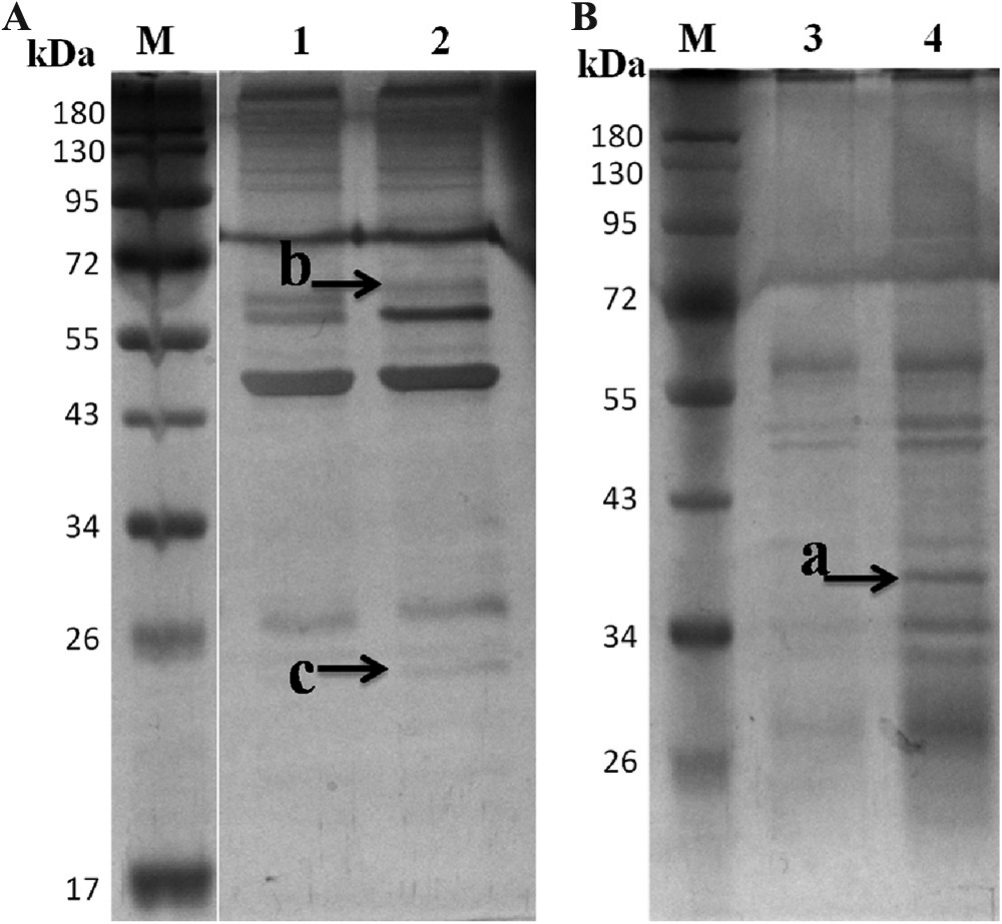
SDS-PAGE of co-IP complex obtained by anti-NSP8 antibody. (A) Total proteins of BmN-NSP8 cells were used for co-IP. Lane M, protein marker; lane 1, immunoprecipitant complex obtained from co-IP conducted with preimmune mouse serum; lane 2, immunoprecipitant complex obtained from co-IP conducted with V5-tag rabbit polyclonal antibody. (B) Total proteins of BmN-NSP8 and BmN-null cells were used for co-IP. Lane 3, immunoprecipitant complex obtained from co-IP carried out with total proteins of BmN-null cells using V5-tag rabbit polyclonal antibody; lane 4, immunoprecipitant complex obtained from co-IP carried out with total proteins of BmN-NSP8 cells using V5-tag rabbit polyclonal antibody.

The total proteins from BmN-NSP8 and BmN-null cells were used for co-IP for screening candidate interacting proteins of NSP8, and the co-IP complex was subjected to sodium dodecyl sulfate-polyacrylamide gel electrophoresis (SDS-PAGE). Compared to the corresponding control, the differential protein bands were recovered for identification by LC-MS/MS (Fig. 8). The identified candidate interacting proteins are shown in Table 1. A total of 25 proteins were identified. Of these, in bands a, b, and c (Fig. 8), 5, 8, and 7 proteins, respectively, were identified with more than two unique peptides. Galectin, endonuclease-reverse transcriptase, mitochondrial prohibitin complex protein 2, elongation factor 1a, translationally controlled tumor protein, and others were implicated in apoptosis. Transformer-2 protein E, DEAD box polypeptide 5 isoform 1, splicing factor 45, arginine/serine-rich splicing factor, and small nuclear ribonucleoprotein polypeptide were all involved in the splicing and processing of RNA. Vasa intronic protein served as a component of RISC.

**TABLE 1:** Mass spectrometry identification of the proteins interacting with BmCPV NSP8^*a*^

Protein no.	Protein name	Mol wt (kDa)/PI	Peptide count/unique peptide count	% Coverage	Function(s) (reference)
a-1	Phosphate carrier 1	39,210/9.01	15/6	10.89	Phosphate ion transmembrane transport (50)
a-2	Mitochondrial prohibitin complex protein 2	33,176/9.7	13/6	17.73	Mitochondrial morphogenesis and apoptosis (51)
a-3	Elongation factor 1-alpha	50,371/9.24	6/3	6.05	Apoptosis (52)
a-4	Transformer-2 protein E	32,388/11.2	6/2	6.12	RNA binding, mRNA splicing (53)
a-5	Phosphoribosyl pyrophosphate synthetase	34,649/6.08	4/2	6.29	Phosphoribosyl pyrophosphate synthesis (54)
a-6	Endonuclease-reverse transcriptase	112,849/9.59	4/1	0.51	Promotes the antivirus immune response by regulating apoptosis (55)
a-7	Non-LTR retrotransposon R1Bmks ORF2 protein	116,256/9.26	3/1	0.48	Non-LTR retrotransposon (56)
a-8	Proliferating cell nuclear antigen	29,035/4.65	2/1	5	DNA replication, DNA repair (57)
b-1	DEAD box polypeptide 5 isoform 1	59,762/9.15	60/16	28.01	RNA processing (58)
b-2	FK506-binding protein	44,677/4.75	48/11	20.65	Protein folding chaperones (59)
b-3	Importin subunit alpha	55,437/5.34	19/7	10.71	Transports protein molecules into the nucleus (60)
b-4	Nucleosome assembly protein	43,045/4.51	10/5	12.77	Chromatin remodeling, cellular responses to viral infection and cell survival (61)
b-5	Glutamate dehydrogenase	61,397/8.36	6/4	5.05	Dehydrogenation of glutamate
b-6	Polyubiquitin	102,423/7.68	5/2	1.97	Selective protein degradation (62)
b-7	Vasa intronic protein	43,807/9.16	4/2	4.37	A component of *Drosophila* RISC (42)
b-8	Seryl-tRNA synthetase	55,545/6.13	2/2	3.44	Seryl-tRNA synthesis
b-9	Cytochrome P450	58,403/8.61	4/1	0.99	Heme proteins (63)
b-10	Splicing factor 45	43,257/5.57	2/1	1.79	RNA splicing (64)
c-1	Heat shock protein hsp20.1	20,138/5.46	25/7	37.08	Responds to heat shock or other environmental stress (65)
c-2	Galectin	24,358/6.3	55/4	17.81	Binds specifically to β-galactoside sugars, apoptosis (66)
c-3	Thiol peroxiredoxin	21,916/6.09	14/4	12.31	Modulates functions of macrophages and dendritic cells (67)
c-4	Translationally controlled tumor protein	19,860/4.66	7/3	15.7	Cell cycle, apoptosis (68)
c-5	Arginine/serine-rich splicing factor	20,174/11.42	7/3	10.23	RNA splicing (69)
c-6	Small nuclear ribonucleoprotein polypeptide	19,649/11.9	4/2	7.49	Alternative splicing (70)
c-7	ADP/ATP translocase	32,891/9.88	2/2	5.33	Exchange of cytosolic ADP and mitochondrial ATP (71)

a LTR, long terminal repeat; ORF, open reading frame.

## DISCUSSION

The sequencing data showed that the length of vsiRNA produced by Dcr-2 differed depending on the host. In fruit flies and mosquitoes of Diptera insects, most vsiRNA is 21 nt in length (38, 39). In Alptera insects (Laodelphax and Homalodisca), vsiRNA is mainly 21 or 22 nt in length (14, 17). In the Hymenoptera (Apis), 22-nt vsiRNAs were mainly produced (40). In the Sf9 cell line of Lepidoptera, the baculovirus-derived vsiRNA was 20 nt in length (41). In the present investigation, the read number for 19-nt vsiRNAs was the highest for BmCPV-derived vsiRNAs in grown BmN cells. The vsiRNA generated in the midgut of larvae infected with BmCPV produced consistent outcomes (16, 33), suggesting that BmCPV genomic dsRNAs were targets of Dcr-2 in BmN cells. The detected vsiRNAs are all derived from the sense strand of viral dsRNAs, indicating that Dcr-2 is biased to cleave the sense strand of BmCPV genomic dsRNA, which may be involved in the distribution of the cleavage signature of Dcr-2. Moreover, BmCPV genomic dsRNA segment S5-derived vsiRNA was not found in infected BmN cells. Most of the vsiRNAs were produced from segments S3 and S4, with 10 vsiRNAs derived from segment S3; of these, 7 had 5′- and 3′-terminal cleavage sites located at nt 2862 to 2865 and nt 2880 to 2882, respectively, indicating that there were hot spots and cold spots for vsiRNA formation among BmCPV genomic dsRNA segments.

Previous studies have shown that the host cells can use their exo-RNAi pathway to resist virus infection, while some viruses can express an RNAi suppressor to counteract the RNAi pathway (20). In the present study, the siRNA-induced RNAi was found to be inhibited in BmN cells by BmCPV infection. It has been reported that the RNAi pathway was inhibited by B2, DCV-1A, and VP3 proteins encoded by FHV, DCV, and CYV, respectively (18–20). In the present study, siRNA-induced RNAi was suppressed by BmCPV NSP8, suggesting that NSP8 was an RNAi suppressor. It was known that RNAi is inhibited by binding B2, DCV-1A, or VP3 proteins to dsRNA to protect dsRNA from being cut by Dcr-2 (18–20). The findings of our investigation revealed that NSP8 could directly attach to viral dsRNAs, suggesting that it competes with Dcr-2 for viral dsRNA binding and protects viral dsRNA from being cut by RNAi machinery. Previous studies have indicated that both the CrPV-1A protein encoded by CrPV and the VP1 protein encoded by Nora virus could counteract/antagonize the activity of Ago2, which is the active component of RISC, through interaction with Ago2 (22, 23). In this study, the interaction between NSP8 and BmAgo2 was confirmed by cellular colocalization and co-IP, suggesting that NSP8 is an antagonizer of BmAgo2. B2 can competitively inhibit the binding of vsiRNA to RISCs (21). Our findings suggested that NSP8 may have an impact on RISC assembly because it interacts with the vasa intronic protein, a component of RISC (42). Dcp2 is a marker for P bodies (36). It has been known that P bodies are dynamic ribonucleoprotein granules composed of translationally repressed mRNAs, mRNA-regulating miRNAs, and proteins related to mRNA decay machinery and play roles in posttranscriptional regulation and RNA metabolism (43). The previous study indicated that BmAgo1 interacts with *B. mori* Dcp2 and that BmAgo1 functions in the miRNA-mediated RNAi pathway (37). Further, recombinant NSP8 was aggregated in the cytoplasm of yeast and colocated with Dcp2. P bodies are evolutionarily conserved in eukaryotes. Therefore, the translation repression controlled by miRNA-mediated RNAi could be regulated by NSP8. Moreover, although our results showed that VP6, NSP5, NSP6, and NSP10 also reduced RNAi, it was not found that these viral proteins could bind to viral dsRNA through a dsRNA pulldown assay, suggesting the involvement of other routes of RNAi suppression.

There are two types of BmCPV infection, namely, persistent infection and pathogenic infection (16, 33). During pathogenic infection, upregulation of both BmAgo2 and BmDcr-2 gene expression and a peak of 20-nt small RNAs were observed. However, BmCPV-derived small RNAs of 20 nt were detected at lower rates in persistently infected larvae. Therefore, BmCPV infection could be regulated by the interaction of NSP8 with RNAi machinery.

In summary, NSP8 encoded by BmCPV was an RNAi suppressor, which could enhance viral proliferation by inhibition of the RNAi pathway through interaction with BmAgo2. Additionally, we discovered certain NSP8 candidate proteins; as a result, additional research into the mechanism and function of NSP8 is required.

### Statistical analysis.

Unless otherwise indicated, statistical tests were conducted using Prism (GraphPad 6.0) software. Data are presented as the mean ± standard error of the mean of results of the independent experiments.

## MATERIALS AND METHODS

### Cells, viruses, and small RNA deep sequencing.

The BmN cells (originating from silkworm ovary) were cultured in TC-100 medium (Gibco BRL, Rockville, MD, USA) supplemented with 10% heat-inactivated fetal bovine serum at 26°C. The preparation of BmCPV stock solution (a lysate of 10^8^ polyhedra/mL) was carried out in accordance with our previous study (44). The small RNA deep sequencing was performed as described in a previous study (16). Briefly, the total RNAs were extracted with a total RNA extraction kit (Qiagen, Valencia, CA, USA) from the BmN cells infected with BmCPV (10 μL stock solution) at 6 days postinfection in accordance with the manufacturer’s manual. The extracted RNA was quantified with a Qubit RNA assay kit (Life Technologies, CA, USA), and the integrity of total RNAs was evaluated using an RNA-6000 nano kit (Agilent Technologies, Santa Clara, CA, USA). The transcriptome library was prepared using a TruSeq RNA sample preparation kit (Illumina, San Diego, USA) according to the instructions. A HiSeq 2500 sequencing instrument (Illumina, San Diego, USA) was used for sequencing.

### Bioinformatics analysis.

The raw data obtained from the Illumina HiSeq 2500 sequencing instrument was analyzed for quality control with the FastQC tool (http://www.bioinformatics.babraham.ac.uk/projects/fastqc/). Low-quality reads, including the reads with 5′ adapter contaminants, the reads without 3′ adapter, the reads without insert, the reads for poly(A), and the reads which are shorter than 15 nt, were removed to obtain clean reads. The obtained clean reads were mapped to the BmCPV genome (GenBank accession no. GU323605, GQ924586, GQ924587, GU323606, GU323606, GQ294469, GQ150538, GQ150539, GQ924588, and GQ924589 for BmCPV genomic dsRNA segments S1 to S10, respectively) and the silkworm reference genome (http://www.ncbi.nlm.nih.gov/genome/?term=silkworm) with bowtie/1.1.2 software by using the mapGenome module of ncPRO-seq v1.6.1. The distribution of vsiRNAs on the viral genomic RNA segments was determined by using Geneious 11.1.2 software (https://www.geneious.com/download/).

### siRNA synthesis.

siLUC (sense, CUUACGCUGAGUACUUCGATT; antisense, UCGAAGUACUCAGCGUAAGTT) and siGFP (sense, GGCUACGUCCAGGAGCGCACC; antisense, UGCGCUCCUGGACGUAGCCUU) siRNAs targeting the firefly luciferase gene (*luc*) and the green fluorescent protein gene (*gfp*), respectively, were synthesized by Gima Corporation (Shanghai, China).

### Dual-Luciferase reporter assay.

A mixture of 2 μg of reporter plasmid pGL3^OTU^ with the *luc* gene driven by *B. mori* ovarian tumor (*Bmotu*) promoter (45), 0.2 μg of *Renilla* luciferase gene reporter plasmid (pRL-TK) (Promega, Carlsbad, CA, USA), and 1 μg of siLUC/siGFP (see Table S1 in the supplemental material) was transfected into BmN cells (10^5^ cells in 1 mL of medium) infected with BmCPV (multiplicity of infection [MOI] of 3) at 48 and 72 h postinfection. In addition, a stock solution of the transfected BmN cells (10^5^ cells in 1 mL of medium) at 48 and 72 h postinfection with BmCPV (MOI of 3), with a mixture of 2 μg of pGL3 (Promega, Carlsbad, CA, USA), 0.2 μg of pRL-TK, and 1 μg of siLUC/siGFP, was used as a control. The cells were collected at 60 h posttransfection and lysed with a passive lysis buffer of the Dual-Luciferase reporter assay system kit (Promega, Madison, WI, USA). One hundred micrograms of protein for each sample was used to determine the activity of luciferase using a GloMax Multi Jr (Promega, Madison, WI, USA).

To assess the effect of viral proteins on siRNA-induced RNAi, 2 μg of pIZT-CS1, pIZT-CS2, pIZT-CS3, pIZT-CS4, pIZT-CS5, pIZT-CS6, pIZT-CS7, pIZT-CS8, pIZT-CS9, or pIZT-CS10 recombinant plasmid expressing VP1, VP2, VP3, VP4, NSP5, VP6, VP7, NSP8, NSP9, or polyhedrin (46), respectively, was cotransfected with 2 μg of pGL3^OTU^, 0.2 μg of pRL-TK, and 1 μg of siLUC/siGFP into 10^5^ BmN cells. The cells collected at 60 h posttransfection were used for the Dual-Luciferase reporter assay.

### Construction of plasmids.

The BmCPV *nsp*8 gene sequence (GenBank accession no. GQ150539) without a stop codon obtained by PCR amplification with primer pairs nsp8-F/nsp8-R (Table S2) from pIZT-CS8 (46) containing the complete cDNA sequence of BmCPV segment S8 was cloned into the SacI/SacII sites of vector pIZT/V5-His (Invitrogen, Frederick, MD, USA) to generate recombinant plasmid pIZT-nsp8, in which the *nsp*8 gene was fused with the V5 tag. The *nsp8* gene amplified using primers nsp8F and nsp8R was subcloned into XmaI/NotI of vector pMVHis to generate plasmid pMVHis-nsp8. The *nsp8* gene amplified using primers nsp8GFP4F and nsp8GFP4R (Table S2) was ligated into SpeI/SalI of pUG34GFP provided by XiaoRong Zhang (N-terminal GFP fusion vector pUG34) (NCBI txid142956) and pUG35-GFP (C-terminal GFP fusion vector pUG35) (NCBI txid142955) vectors to construct plasmids pUG34GFP-nsp8 and pUG35-nsp8-GFP, respectively.

### Construction of transformed BmN cells.

The cultured BmN cells were transfected with the recombinant plasmid pIZT-nsp8 using Lipofectin (Roche, Indianapolis, Germany). The transfected cells were screened continuously for a month with zeocin (final concentration, 200 μg mL^−1^) at 3 days posttransfection to generate transformed BmN-nsp8 cells expressing the *nsp8* gene. In the meantime, pIZT/V5-His was used to generate transformed BmN-null cells (as control cells).

### dsRNA pulldown assay and LC-MS/MS.

Extraction of BmCPV genomic dsRNA from polyhedra was performed as previously described (25), and the purified BmCPV genomic dsRNAs were labeled with biotin using a Pierce RNA 3′-end desthiobiotinylation kit (Thermo; catalog no. 20163) according to the manual’s instructions. Biotin-labeled dsRNAs were incubated with total extracted proteins from BmCPV-infected midgut at 4°C for 2 h. Subsequently, the mixture was incubated with 20 μL of streptavidin agarose (Thermo Scientific, USA; catalog no. 20349) for another 2 h at 4°C with slow agitation, followed by washing with lysis buffer (Tris-HCl, 0.0788 g; EDTA, 0.0146 g; NaCl, 0.7305 g; Triton X-100, 250 μL) to reduce the nonspecific binding proteins. After centrifugation at 12,000 × *g* for 10 min, the precipitates were separated by SDS-PAGE. The protein bands were visualized by silver staining. Meantime, dsRNA pulldown performed with the BmCPV dsRNAs without biotin was used as a control (47). The differential band from the biotin-labeled BmCPV dsRNA group was extracted and identified by LC-MS/MS (Shanghai Lu Ming Biological Technology Co., Ltd.).

### SDS-PAGE and Western blotting.

The total proteins extracted from the transformed BmN cells expressing a viral protein or the transformed BmN-null cells were mixed with 2× SDS loading buffer (0.1 mol L^−1^ Tris-Cl, 0.2 mol L^−1^ dithiothreitol, 4% SDS, 20% glycerol, 0.2% bromophenol blue, 4% β-mercaptoethanol) and incubated in a water bath at 100°C for 5 min. After centrifugation at 12,000 × g for 3 min, the supernatant was subjected to SDS-PAGE with a 5% stacking gel and a 12% separating gel, respectively. The separated proteins on the gel were visualized with Coomassie brilliant blue R250 staining. After the proteins on the gel were transferred to a polyvinylidene difluoride membrane, Western blotting was conducted by using a rabbit anti-V5 polyclonal antibody (CWBiotech, Beijing, China) as the primary antibody and a horseradish peroxidase (HRP)-labeled goat anti-rabbit IgG as the secondary antibody (Servicebio, Wuhan, China). In addition, β-tubulin, used as a reference, was detected by Western blotting with Tubulin βmAb (Bioleaf, Shanghai, China) and HRP-labeled goat anti-mouse IgG (Servicebio, Wuhan, China). The Western blotting image was analyzed by grayscale scanning with ImageJ software (https://imagej.en.softonic.com/?ex=BB-682.0#).

### Real-time PCR.

The transformed BmN cells (10^5^ cells mL^−1^, 1 mL) expressing a viral protein gene or transformed BmN-null cells (10^5^ cells mL^−1^, 1 mL) were infected with 20 μL of BmCPV stock solution, the total RNAs were extracted from the cells at 24 h postinfection by using TransScript one-step genomic DNA (gDNA) removal (TransGen Biotech, Beijing, China), and the cDNA was synthesized with a cDNA synthesis supermix kit (TransGen Biotech, Beijing, China). Subsequently, the cDNA was used as a template, and the expression level of the BmCPV *vp1* gene was determined by real-time PCR with specific primer pairs (Table S3) using TransStart tip green real-time PCR supermix (TransGen Biotech, Beijing, China). The housekeeping gene translation initiation factor 4A (*TIF-4A*) of *B. mori* was used as an internal control for normalization. The 2^–ΔΔ^*^CT^* method was used to calculate the relative expression level of the target gene (48). Each experiment was repeated three times.

### Urea-PAGE.

The small RNAs in total were extracted from the midgut of BmCPV-infected silkworm at 1 to 6 days postinfection, and the BmCPV-infected BmN cells were transfected with pIZT-NSP8 according to the instructions of the RNAiso kit for small RNA extract (TaKaRa, catalog no. 9753). Ten microliters of the small RNAs in total was subjected to 15% urea-PAGE, and the 5.8S rRNA was used as a reference as described in a previous study (49). Uninfected silkworm midgut and BmCPV-infected BmN cells transfected with pIZT-V5/His were used as controls.

### Polyclonal antibody preparation.

The coding sequence of the BmCPV *nsp*8 gene (GenBank accession no. GQ150539) and the partial coding sequence (nt 380 to 802) of the *BmAgo2* gene (GenBank accession no. NM_001043530.2) obtained by PCR with primer pair BmAgo2F/BmAgo2R (Table S2) were, respectively, cloned into the NcoI/XhoI and BamHI/XhoI sites of the expression vector pET-28a(+) (Novagen, Darmstadt, Germany) to generate recombinant plasmids pET-28a-nsp8 and pET-28a-BmAgo2. The purified recombinant NSP8 protein and recombinant truncated BmAgo2 protein expressed in Escherichia coli strain Transetta (DE3) were used to immunize ICR mice (Soochow University, Suzhou, China) by subcutaneous injection. The specificity of the prepared antibody was then identified by Western blotting.

### Immunofluorescence assay.

BmCPV-infected BmN cells at 48 h postinfection and BmN-NSP8 and BmN-null transformed cells, each at 10^4^, were washed with 1× phosphate-buffered saline (PBS) three times and fixed with 4% paraformaldehyde for 5 min. Immunofluorescence assays were carried out using the prepared mouse anti-NSP8 antibody or the rabbit anti-V5 polyclonal antibody (CWBiotech, Beijing, China) as the primary antibody and fluorescein isothiocyanate (FITC)-conjugated goat anti-mouse IgG(H+L) (Proteintech, USA; catalog no SA00003-1) and Cy3-conjugated goat anti-rabbit IgG(H+L) (Servicebio, Wuhan, China) as the secondary antibodies for determining the subcellular localization of the NSP8 protein. To determine the colocalization of NSP8 with BmAgo2 in the BmN-NSP8 transformed cells, immunofluorescence tests were performed using rabbit anti-V5 polyclonal antibody and mouse anti-BmAgo2 as the primary antibodies and Cy3-conjugated goat anti-rabbit IgG(H+L) and Cy5-conjugated goat anti-mouse IgG (Servicebio, Wuhan, China) as the secondary antibodies. The cell nucleus was labeled with DAPI (1:1,000). Images were collected with a Leica TCS SP8 confocal microscope (Leica Microsystems, Mannheim, Germany).

### Localization of NSP8 in yeast.

Wild-type yeast strain BY4743 was cotransfected with 2 μg of plasmid pUG34-GFP-nsp8/pUG35-nsp8-GFP encoding GFP-nsp8/nsp8-GFP driven by the *MET25* promoter and 2 μg of plasmid encoding the mRNA decapping protein (Dcp) fused with a red fluorescent protein (Dcp2-RFP) (72). Transformants were grown on MY medium containing leucine, uracil, and methionine at 30°C for 3 days. After that, the positive colonies were inoculated at 30°C in MY liquid medium containing leucine, uracil, and methionine overnight. The cultured cells were washed twice with double-distilled water and resuspended in MY liquid medium without methionine to induce the expression of NSP8 for an additional 1 h at 30°C. GFP signal (excitation, 488 nm; emission, 520 nm) and RFP signal (excitation, 561 nm; emission, 575 nm) were observed under a Zeiss Imager confocal microscope.

### Co-IP.

The total protein was extracted from the BmN-NSP8 cells and incubated with prepared mouse anti-NSP8 antibody or rabbit anti-V5 polyclonal antibody at 4°C overnight. The protein-antibody complex was incubated with protein A+G (CWBio, China; catalog no. CW0349S) at 4°C for 2 h. The complex was washed with 1× PBS 6 times, centrifuged to retain the precipitate, and incubated in boiled water for 10 min with 5× SDS loading buffer (Beyotime, China; catalog no. P0015). After centrifugation, the supernatant was used for Western blotting. The infected and uninfected protein extracted from the silkworm midgut was extracted for incubation with rabbit V5 antibody or IgG, and after incubation with protein A+G, the protein was separated by SDS-PAGE. The differential band was extracted and identified by LC-MS/MS (Shanghai Lu Ming Biological Technology Co., Ltd.).

### Data availability.

The raw data of small RNA deep sequencing have been deposited in the NCBI database under accession number SRR21047179.
