# Supplementary material for: Interaction between Bombyx mori Cytoplasmic Polyhedrosis Virus NSP8 and BmAgo2 Inhibits RNA Interference and Enhances Virus Proliferation
Source: Microbiol Spectr. 2023 Jun 21;11(4):e04938-22. doi: 10.1128/spectrum.04938-22 (PMC10434170; doi:10.1128/spectrum.04938-22)
Supplement: Supplemental file 1 — Supplemental material. Download spectrum.04938-22-s0001.pdf, PDF file, 0.3 MB [file spectrum.04938-22-s0001.pdf]

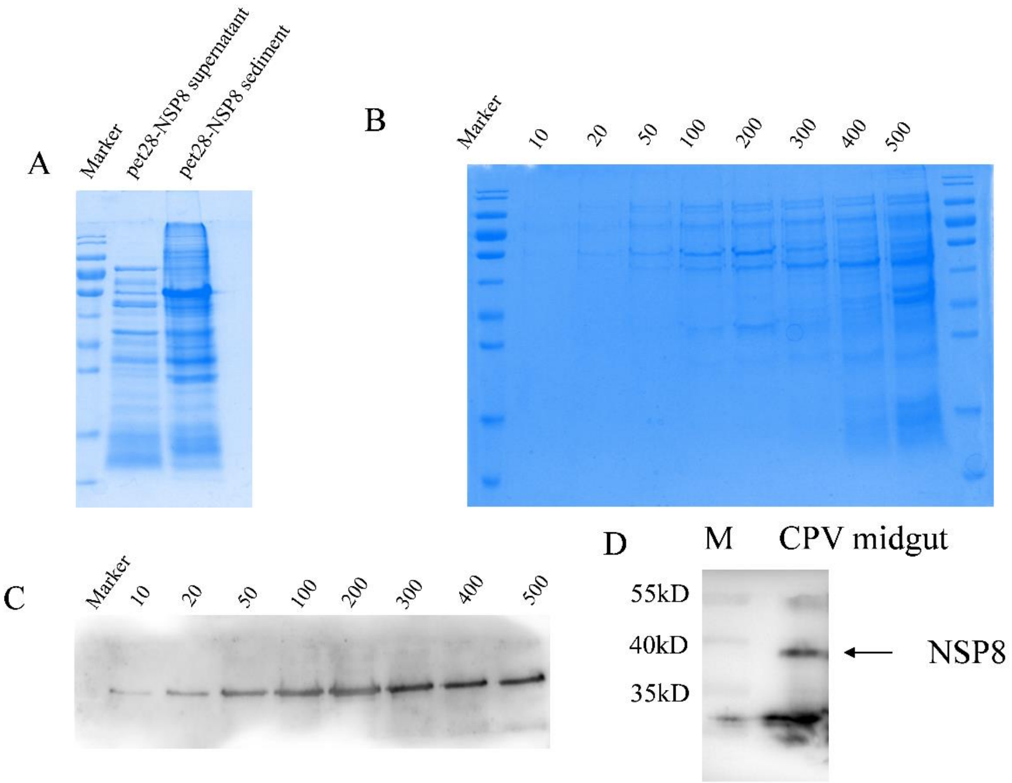

Figure S1 **Specificity of the prepared mouse anti-BmNSP8 antibody was detected with Western blotting.** (A) The pET28-NSP8 transformed into *E. coli* BL21 was collected and ultrasonic crushed with PBS, the supematant and sediment was separated by SDS-page. (B) The pet28-NSP8 sediment was further crushed with GUNTA-0 (0.5M Tris-HCl pH7.9; 0.5M Nacl; 7M Urea; 10% Glycerinum) and eluted with imidazole (10mM; 20mM; 50mM; 100mM; 200mM; 300mM; 400mM; 500mM). (C) The elution volume was confirmed by the prepared mouse anti-NSP8 antibody. (D) The prepared mouse anti-NSP8 antibody was used to detect the NSP8 protein expressed in the BmCPV infected BmN cells. HRP-labeled goat anti-mouse IgG was used as the second antibody.

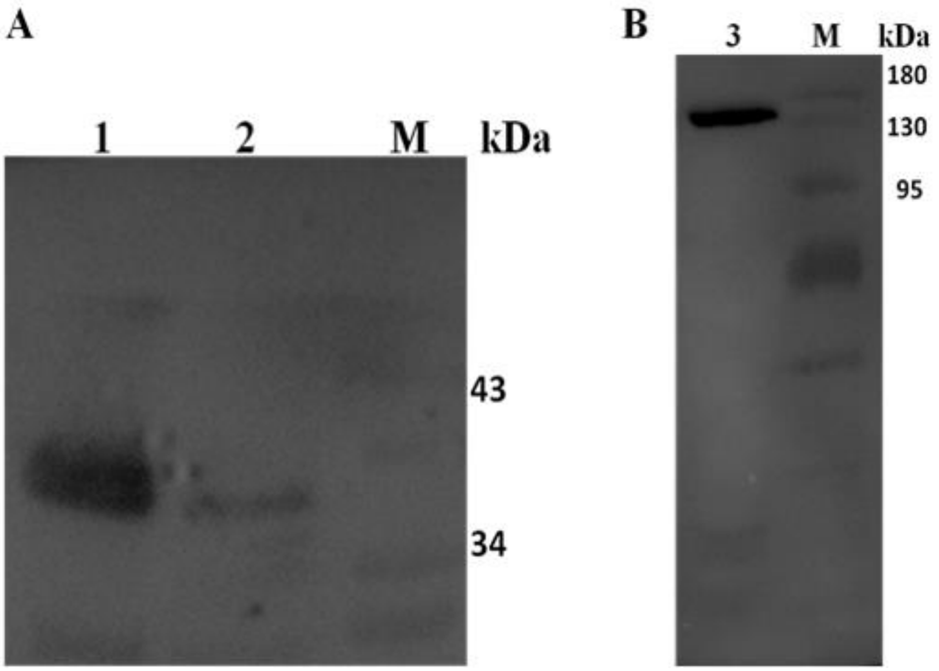

Figure S2 Specificity of the prepared mouse anti-BmAgo2 antibody was detected with Western blotting.

(A) The prepared mouse anti-BmAgo2 antibody was used to detect the recombinant truncated BmAgo2 protein expressed in *E. coli* BL21 transformed with pET28a-BmAgo2. Lane M, protein marker; lane 1, *E. coli* BL21 transformed with pET28a-BmAgo2; lane 2, *E. coli* BL21 transformed with pET28a(+). (B) The prepared mouse anti-BmAgo2 antibody was used to detect the BmAgo2 protein expressed in the BmN cells. Lane 3, the cultured BmN cell. HRP-labeled goat anti-mouse IgG was used as the second antibody.

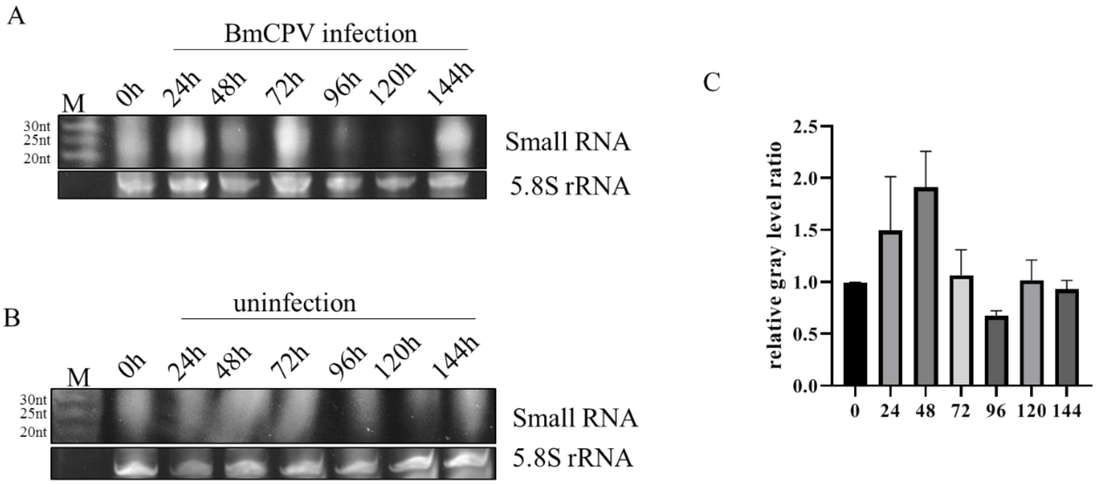

Figure S3 The small RNAs produced in the midgut at 24-144h post-infection with BmCPV.

The new molted larvae of 5th instar silkworm were infected BmCPV by feeding mulberry leaves coated with 10^6 polyhedra/mL for 8h. The total small RNAs were extracted from the silkworm midgut at 1-6 days post-infection following the instructions of RNAiso kit (Takara, 9753) for small RNA extraction. 10μl of total RNAs was separated by 15% Urea-PAGE. 5.8S rRNA was used as a reference. A, Urea-PAGE of total small RNAs extracted from the BmCPV-infected silkworm midgut; B, Urea-PAGE of total small RNAs extracted from the uninfected silkworm midgut; C, Results of the grayscale of small RNAs band on Urea-PAGE gel. The vertical axis represents the gray level ratio between small RNAs and 5.8S rRNA relative to their corresponding controls.

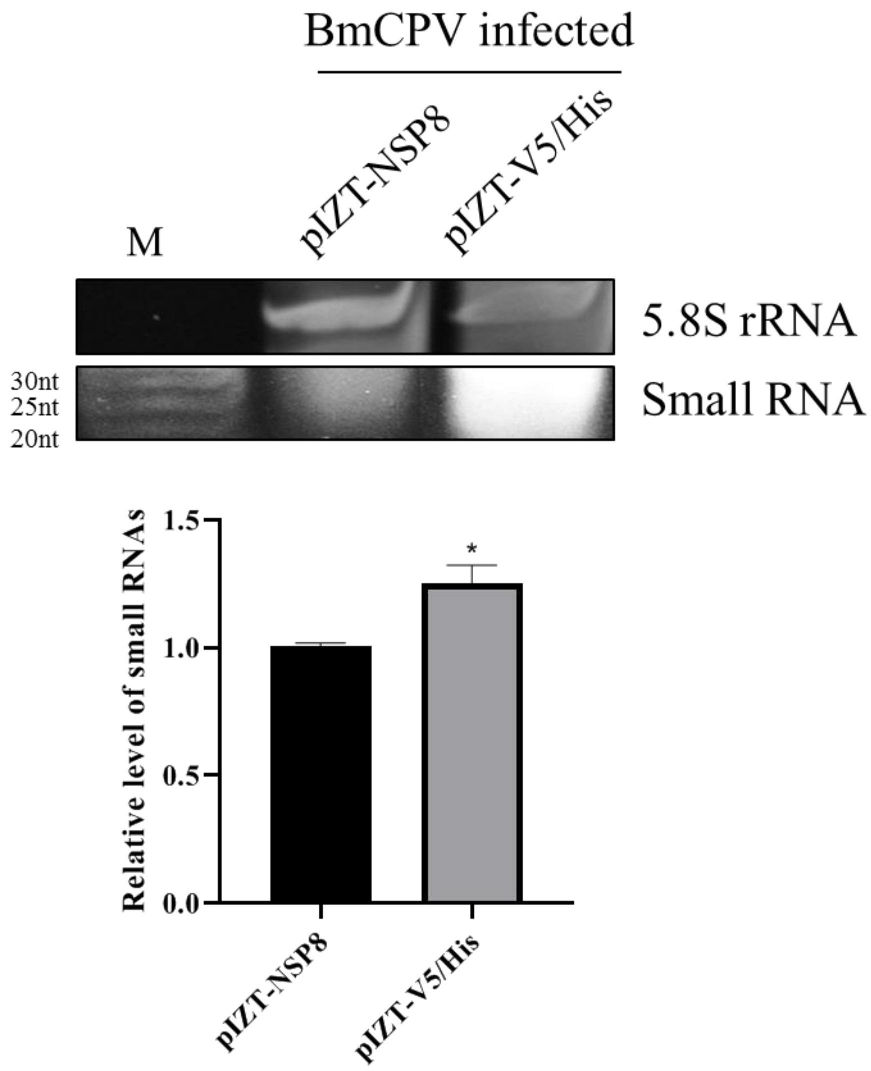

Figure S4 Effect of overexpression of NSP8 on the formation of total small RNAs in BmN cells infected with BmCPV

10^6 of BmN cells were infected with BmCPV (MOI=3), 48h later, the cells were transfected with 2μg of pIZT-NSP8, meanwhile, the BmCPV-infected cells transfected with empty vector pIZT-V5/His were used as control. The total small RNAs were extracted from the treated BmN cells at 48h post-transfection according to the instructions of RNAiso kit (Takara, 9753) for small RNAs extraction, 10μl of total small RNAs was subjected to 15% Urea-PAGE and the 5.8S rRNA was used as an reference. Upper, Urea-PAGE of total small RNAs extracted from the treated BmN cells; Below, Results of the grayscale of small RNAs band on Urea-PAGE gel.
